# Formation of benign tumors by stem cell deregulation

**DOI:** 10.1371/journal.pgen.1010434

**Published:** 2022-10-27

**Authors:** Matthieu Valet, Patrick Narbonne

**Affiliations:** Département de biologie médicale, Université du Québec à Trois-Rivières, Trois-Rivières, Québec, Canada; Brigham and Women’s Hospital, UNITED STATES

## Abstract

Within living organisms, stem cells respond to various cues, including to niche signals and growth factors. Niche signals originate from the stem cell’s microenvironment and promote the undifferentiated state by preventing differentiation, allowing for stem cell self-renewal. On the other hand, growth factors promote stem cell growth and proliferation, while their sources comprise of a systemic input reflecting the animal’s nutritional and metabolic status, and a localized, homeostatic feedback signal from the tissue that the stem cells serve. That homeostatic signal prevents unnecessary stem cell proliferation when the corresponding differentiated tissues already have optimal cell contents. Here, we recapitulate progresses made in our understanding of in vivo stem cell regulation, largely using simple models, and draw the conclusion that 2 types of stem cell deregulations can provoke the formation of benign tumors. Namely, constitutive niche signaling promotes the formation of undifferentiated “stem cell” tumors, while defective homeostatic signaling leads to the formation of differentiated tumors. Finally, we provide evidence that these general principles may be conserved in mammals and as such, may underlie benign tumor formation in humans, while benign tumors can evolve into cancer.

Our tissues have usually just the right number of cells to optimally fulfil their function. Not enough cells within a tissue can lead to dysfunction, while too many cells may form a tumor. Tumors are distinct from tissue hyperplasia, which is reversible and developmentally or environmentally triggered, and in which the extra cells are well organized within the tissue. Tumors, on the other hand, are disorganized and display continued growth. Tumors may be initially benign and consist of disorganized cells that lack the ability to invade neighboring tissues or metastasize. Hence, benign tumors are not cancerous and, depending on where they arise, they are usually not an immediate life-threatening problem. However, benign tumors have the potential to become malignant [1–4] and, as such, their formation may be considered a key early step in carcinogenesis. Yet, a clear rationale explaining how benign tumors may arise has, to our knowledge, never been formulated. Most cells within early-stage benign tumors are typically well differentiated, albeit in rarer cases, they can be undifferentiated [5–7]. Here, we first recapitulate advances made in our basic understanding of in vivo stem cell (SC) regulation, while many of these principles were discovered in simple models. Interestingly, the data suggest that benign tumors, undifferentiated and differentiated, each develop from a different type of SC deregulation. Namely, constitutive niche signaling causes the formation of undifferentiated tumors, while defective homeostatic signaling leads to the formation of differentiated tumors. These emerging notions are supported by the mammalian literature, where available, and may thus be highly conserved.

## Stem cell regulation

SCs have a unique dual potential, showing both the capacities to self-renew, and to differentiate into other cell types. Since the original demonstration of these capacities in hematopoietic SCs [8], several other SC types have been discovered and characterized, such as the mammalian pluripotent embryonic SCs (ESCs) [9], and various adult SCs (also known as tissue specific, tissue resident, or multipotent SCs). Our understanding of in vivo SC regulation was however hindered by their limited accessibility in most systems [10]. As such, the landmark introduction of in vitro SC culture revolutionized SC research by facilitating SC expansion, manipulation, and microscopic observation [9]. Yet, SCs respond to various signals within a living organism. For example, the existence of a specialized microenvironment preventing hematopoietic SC maturation was hypothesized in 1978 [11], but could not be readily demonstrated in this complex system. A range of SCs were thus discovered and characterized in simple invertebrate models that allow the effective combination of genetic, cell biological, and molecular approaches, such as *Drosophila* [12,13] and *Caenorhabditis elegans* [14]. This allowed for the discovery of the SC niche, niche signaling, and more recently, of additional mechanisms regulating SC proliferation rates. In the next 2 sections, we briefly recapitulate how SCs are regulated by niche signaling and growth factors in vivo, through highly conserved basic principles [15–18].

## Regulation of stem cell fate by niche signaling

**Fig 1:**
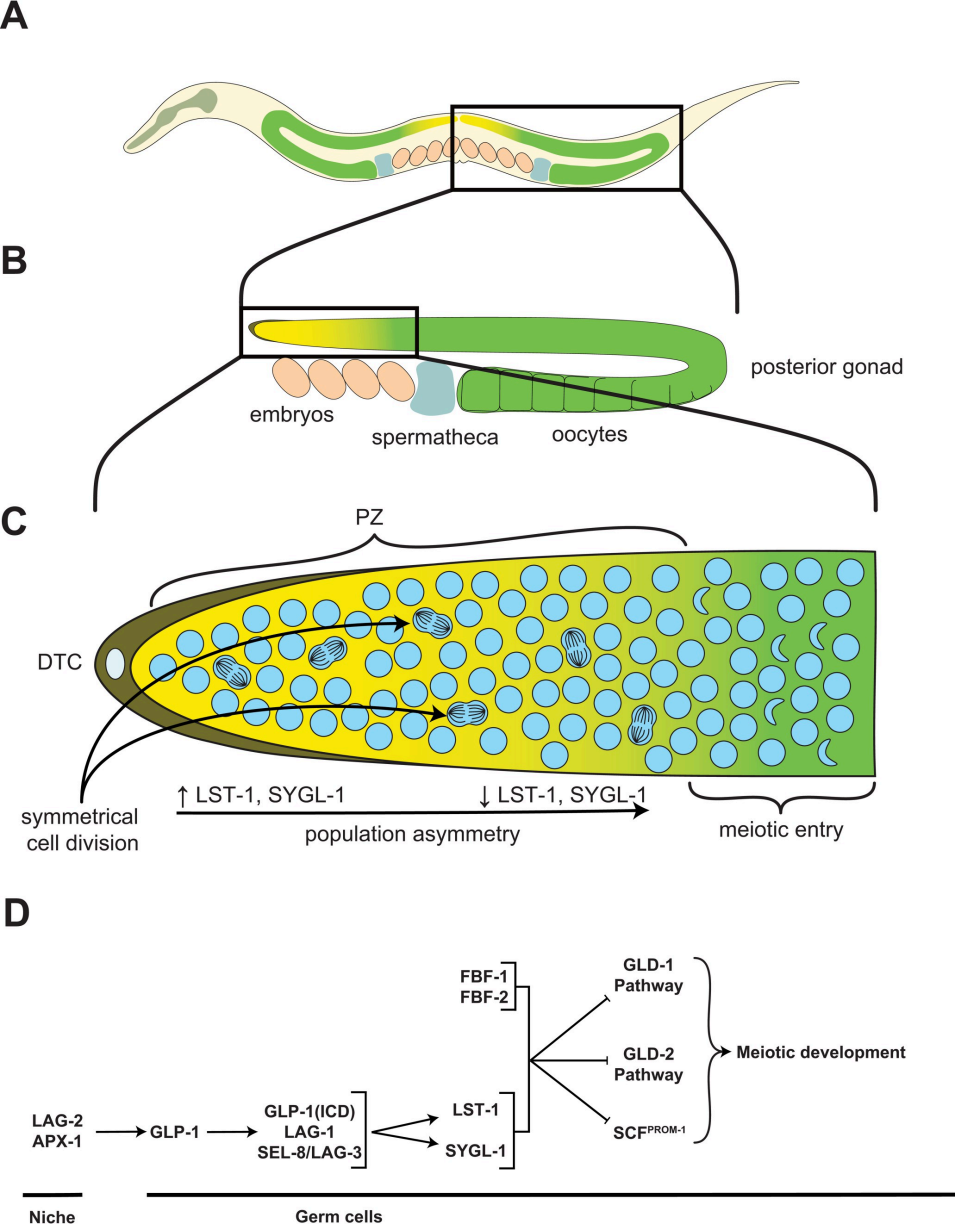
The *C*. *elegans* GSC system. ** (A)** Diagram of an adult *C*. *elegans* hermaphrodite, highlighting its 2 symmetrical U-shaped gonad arms (adapted from *www.wormatlas.org*). **(B)** Magnified schematic of the animal’s posterior gonad arm. **(C)** Further magnified schematic of the distal gonad, revealing the niche cell (DTC; dark yellow), the progenitor zone (PZ; light yellow), and meiotic entry zone (green). GSCs undergo symmetric divisions since daughters have a similar fate potential. Yet, a population asymmetry becomes apparent through a gradient of GLP-1 activity, and levels of its transcriptional targets LST-1 and SYGL-1, that fades proximally, until a threshold is reached and cells commit to meiosis [56,64,121,122]. This is apparent as their nuclei adopt the shape of a crescent. **(D)** Simplified genetic model for niche signaling. The DTC acts as the GSC niche by expressing the Notch ligands LAG-2 and APX-1, which activate the receptor GLP-1 at the surface of GSCs. When GLP-1 is activated, its ICD is cleaved and serves as a co-activator for the transcription factor LAG-1, which together with LAG-3 and SEL-8, binds to and activates the transcription of 2 main target genes, *lst-1* and *sygl-1* [56,64,121,122]. LST-1 and SYGL-1 then work together with FBF-1 and FBF-2, to repress 3 largely parallel, but partly redundant pathways (GLD-1, GLD-2, and SCF^PROM-1^) that together promote differentiation. Simplified from [54]. DTC, distal tip cell; GSC, germline stem cell; ICD, intracellular domain.

SC niches are local tissue microenvironments that support SC maintenance through signaling [16]. The niche often involves multiple cell types and signaling pathways in mammals [16,19], but in its simplest configuration, it may exist as a single cell that prevents neighboring SCs from differentiating via the expression of a single factor. The *C*. *elegans* germline SCs (GSCs) form 1 such simple example, where a single-celled niche called the distal tip cell (DTC), prevents GSC differentiation through activating their Notch receptors (Fig 1). In this system, when the DTC was ablated, or when Notch signaling was blocked, all GSCs were quickly lost to differentiation [14,20,21]. From thereon, niche signaling has been regarded as the signal(s) that instructs SC fate, preventing SC differentiation. Several mammalian niches have later been described, including those of the hematopoietic, hair follicle, skin, lungs, and intestinal SCs; however, these are far more complex, involving multiple cell types and signaling molecules [22–25]. Despite this complexity, the primary role of the mammalian niche is to maintain SCs in an undifferentiated state, as in *C*. *elegans* and *Drosophila* [16]. However, we note that “niche signaling” in mammalian systems is sometimes used in reference to “any signal that regulates SC function,” including regulation by growth factors. In this review, we consider growth factors that primarily influence SC growth/proliferation as a form of signaling to the SCs that is distinct from niche signaling (see below).

That SCs readily initiate differentiation in the absence of niche signals further implies that SCs are “primed” for differentiation and are essentially waiting for niche signals to weaken to launch their differentiation program. This naturally happens when SCs move away from the niche. This may occur in a predictable manner when SCs primarily divide asymmetrically to give rise after each division to another SC that remains attached to the niche, and a daughter cell that is born away from the niche and differentiates [26]. In other systems however, including the *C*. *elegans* GSCs, niche signaling may have a broader range, and SCs may exist as a homogeneous pool in which SCs divide symmetrically until some of their progenies move away from the niche by neutral drift and differentiate [15,27].

## Regulation of stem cell proliferation rates

**Fig 2:**
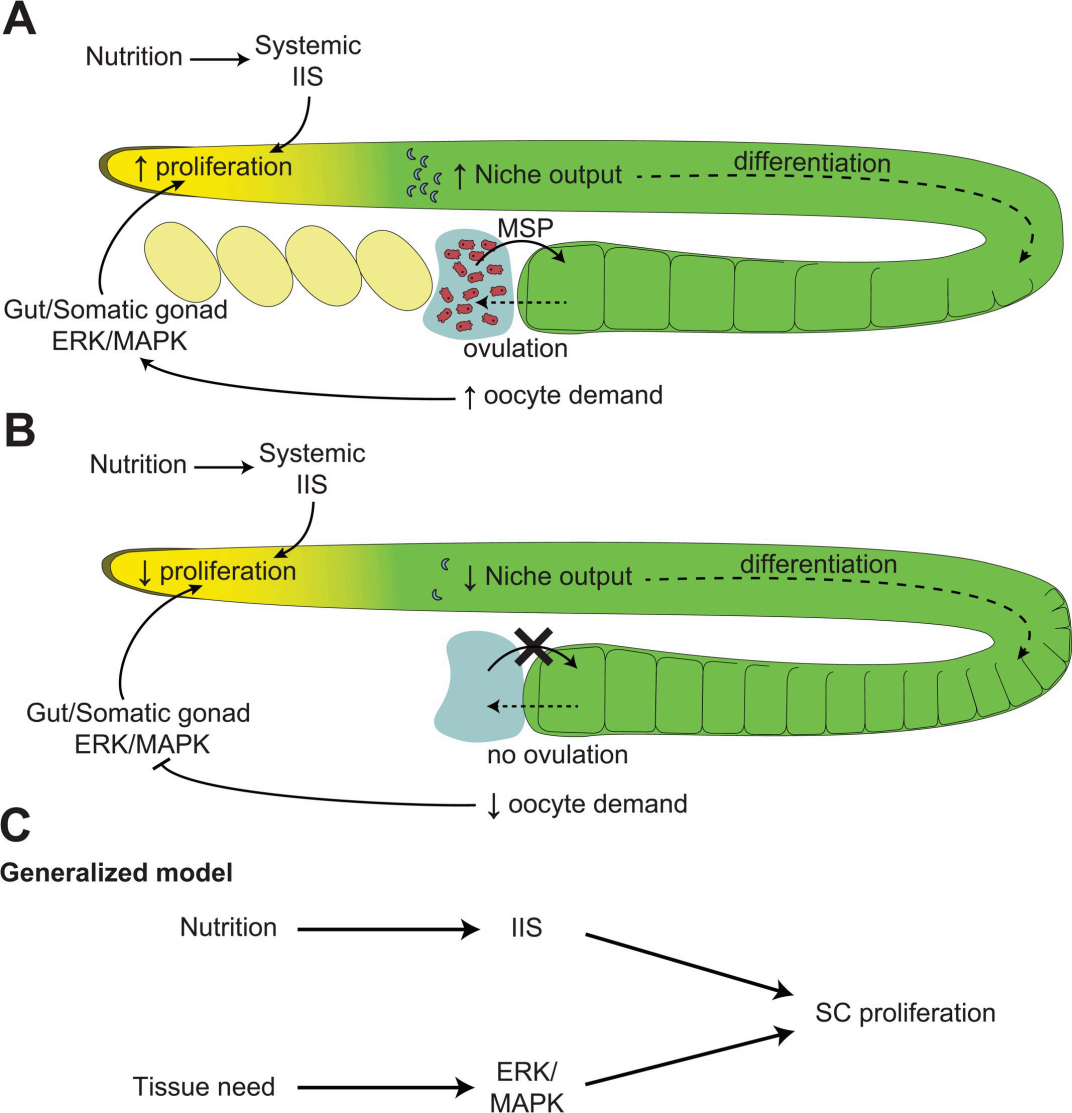
SC proliferation regulatory mechanisms. **(A)** Schematic representation of the 2 known mechanisms that regulate GSC proliferation in young adult *C*. *elegans* hermaphrodites. The nutritional status systemically regulates GSC proliferation through activating IIS. In this case, the insulin receptor DAF-2 and its main downstream transcriptional target DAF-16/FOXO both act within the germline to influence GSC proliferation [33,35]. In parallel, the presence of sperm, through release of MSPs, promotes oocyte ovulation, maintaining a high oocyte demand. This presumably activates MPK-1/ERK in the animal’s gut and/or somatic gonad, which nonautonomously promotes GSC proliferation, and sustains an elevated niche output and oocyte production [41]. **(B)** When sperm is depleted, ovulation is suppressed and oocytes initially accumulate in the proximal gonad, thereby reducing the demand for new oocytes. This is expected to suppress MPK-1/ERK signaling in the gut and/or somatic gonad, thereby slowing down GSC proliferation and oocyte production. **(C)** A generalized model for the regulation of SC proliferation by nutrition and tissue demand. Note that while the function of IIS thus far appears well conserved [18], ERK/MAPK signaling could be replaced by other pathways in different SC types or organisms. Additional regulatory mechanisms may also exist. GSC, germline stem cell; IIS, insulin-like/IGF-1 signaling; MSP, major sperm protein; SC, stem cell.

When an SC lies within its niche and is thus instructed to remain undifferentiated, it can proliferate or remain quiescent. Growth factors promote SC proliferation, while their absence promotes quiescence. At least 2 separate kinds of growth factors are known to stimulate SC growth and proliferation. A systemic signal was first shown to stimulate SC proliferation in order to match its rate to the nutritional status of the animal, which reflects on the availability of both basic nutrients and energy, such that SCs will generally proliferate more in a well-nourished animal and less in a starved one [17,18,28–30]. A more localized signal can further couple the proliferation rates of specific SC populations within an organism to the needs for new differentiated cells in the tissue they serve (Fig 2). These 2 mechanisms are individually discussed in the next subsections.

### Systemic regulation of stem cell proliferation downstream of nutrition

Niche signals were rapidly recognized to instruct SC fate, but what controlled the rate of SC proliferation remained unclear until it was noticed that the proliferation of *Drosophila* GSCs responded to the animal’s nutritional status [28]. Indeed, following nutrition, insulin-like peptides are secreted from the animal’s nervous system and promote GSC proliferation, in both males and females [29,31]. Nutrition and systemically released insulin-like peptides also support the proliferation of *Drosophila* intestinal SCs, neuroblasts, and hematopoietic progenitors [18]. Equally in *C*. *elegans*, insulin-like/IGF-1 signaling (IIS) is activated in response to nutrition and systemically promotes GSC proliferation [17,32–34]. In this system, the sole insulin-like receptor DAF-2 cell-autonomously promotes GSC proliferation through inhibiting the forkhead transcription factor DAF-16/FOXO [33,35]. In mammals, dietary changes such as calorie restriction, ketogenic, or high-fat diets also systemically influence the growth/proliferation of mammalian SCs, although the responses to perturbations are often tissue specific [17,18,30,36,37]. In addition to diet, other systemic factors may independently influence the proliferation of certain SC populations, including those linked to aging, immune responses, and circadian rhythms.

### Localized regulation of stem cell proliferation by tissue homeostasis

An animal’s nutritional status however does not fully explain the proliferative behavior of all its SCs. Indeed, not only do the SCs of each tissue divide at different rates [38], but a disparity can sometimes be observed across separate populations of the same SC type. In the *C*. *elegans* adult hermaphrodite for example, GSC proliferation rates are coupled to nutrition and IIS, but also to the demand for oocytes, their terminally differentiated progenies. When the animal’s spermatheca is empty, mature oocytes are no longer fertilized and begin to accumulate in the proximal gonad. This triggers a feedback signal that suppresses GSC proliferation at the distal end, to pause the production of new oocytes (Fig 2B) [34,39,40]. This homeostatic signal was shown to take place independently within each of the animal’s 2 gonad arms [34].

It was also realized that GSC proliferation plummets in spermless *C*. *elegans* hermaphrodites despite their sustained feeding and systemically activated IIS [34,35]. Genetic and epistasis analyses revealed that ERK/MAPK signaling promoted GSC proliferation in parallel to IIS and that the localized homeostatic regulation relied on ERK/MAPK suppression to inhibit GSC proliferation [35,41]. As such, nutrition through IIS and homeostatic signaling through ERK/MAPK act in concert to promote the highest levels of GSC proliferation in *C*. *elegans*. If either ERK/MAPK or IIS decreases, GSC proliferation decreases, but it completely stalls only when both pathways are simultaneously inactivated (Fig 2C).

Localized homeostatic SC regulation is conserved in more complex systems. For example, *Drosophila* midgut intestinal SC proliferation was shown to be stimulated both by IIS downstream of nutrition and by homeostatic cytokines released by damaged enterocytes. These cytokines activate EGFR and JAK/STAT signaling more locally within intestinal SCs to promote enterocyte replacement [7,18,42]. In mammals, muscle SCs are normally quiescent throughout adult life, yet they resume proliferation following a muscle injury to participate in repair [43]. Although, to our knowledge, this has not been specifically demonstrated, it is expected that only the muscle SCs located close to the injury resume proliferation. It seems somewhat inconceivable that all muscle SCs across all muscles would re-enter proliferation following a localized muscle injury. Therefore, localized homeostatic control of SC proliferation must be a widely spread phenomenon, applicable to several types of SCs that serve solid tissues such as the skin, muscle, lungs, and the gastrointestinal epithelium. Homeostatic SC regulation also certainly occurs in liquid tissues, such as the blood and lymph of mammals (e.g., homeostatic regulation of erythropoiesis by erythropoietin [44]), though it more likely acts systemically within these tissues. Regulation of SC proliferation by additional mechanisms, localized or systemic, may take place, including from cues related to developmental stage, aging, inflammation, immunity, and/or stress. For example, SC division rates were shown to significantly decrease with age in *C*. *elegans* [34,45], *Drosophila* [46,47], and in the human colon [48].

## Niche signaling boundaries confine undifferentiated stem cells

**Fig 3:**
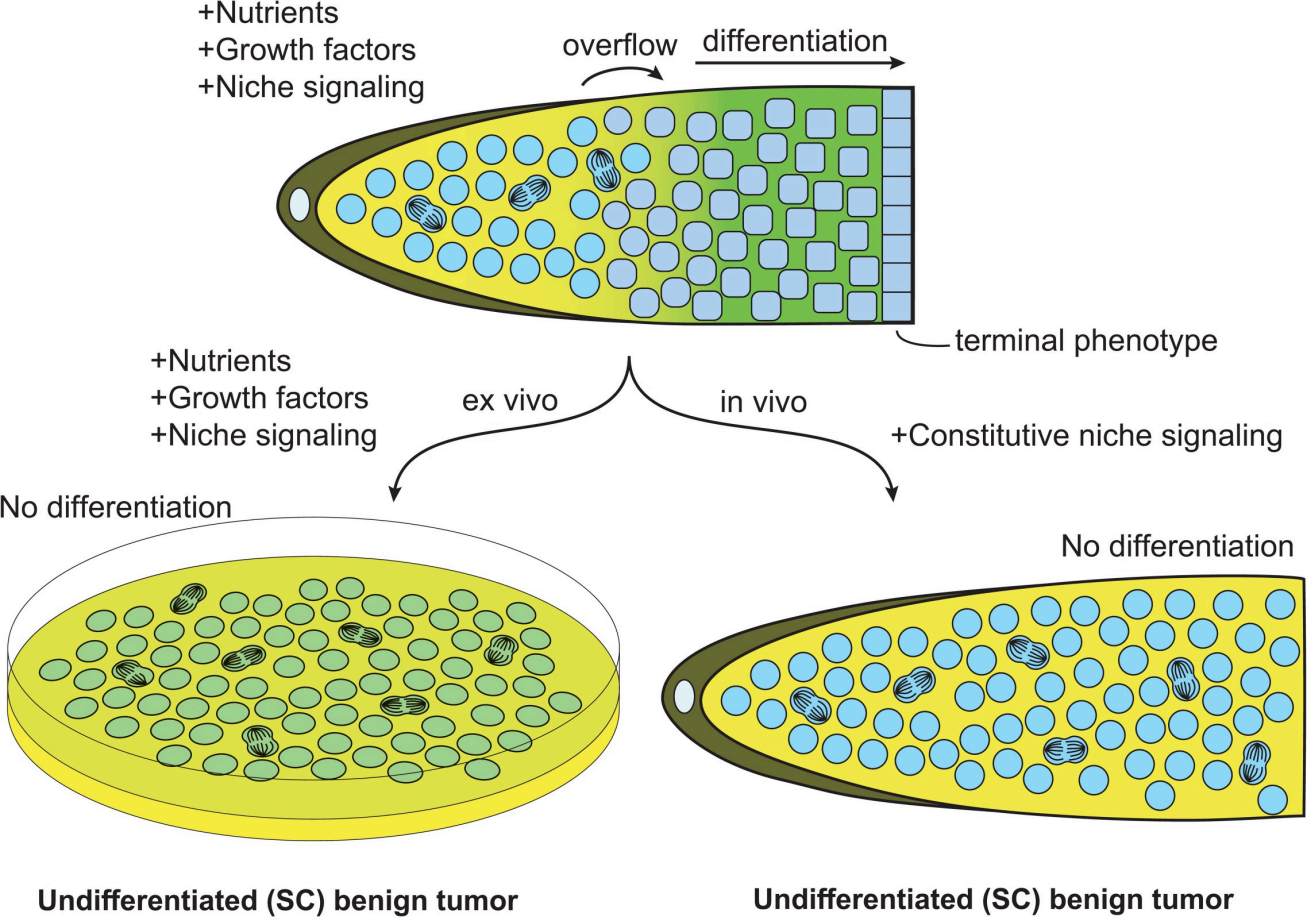
Undifferentiated SC tumors in vitro and in vivo. Top, graphical representation of a simple in vivo SC system or tissue. Left, extracted SCs grown in vitro form an ever-expanding undifferentiated benign tumor. Right, in vivo, undifferentiated benign tumors can occur when a mutation constitutively activates niche signaling. SC, stem cell.

The SC basic properties and regulatory mechanisms, as defined above, are such that when SCs are isolated from an animal and put in a culture dish, they essentially require 3 elements to grow, undergo self-renewing proliferation, and expand in numbers to eventually fill that dish. Let us use the mammalian ESCs to illustrate this point. ESCs extracted from an embryo first need to continue receiving niche signaling in order to remain undifferentiated; otherwise, they would launch their default differentiation program. In culture, niche signaling was originally recreated by coating the dish with a layer of inactivated mouse embryonic fibroblasts (MEFs) [9]. Nowadays, niche signaling is more conveniently and efficiently replaced by the 2iL cocktail (containing mitogen-activated protein kinase kinase (MEK) and glycogen synthase kinase (GSK3) inhibitors (2i), in addition to the leukemia inhibitory factor (LIF, L) cytokine) to the medium, which acts to prevent differentiation and promote the naïve state [49–53]. For ESCs to grow and proliferate, they further require basic nutrients to synthesize new nucleic acids and proteins, as well as growth factors to promote nutrient utilization. These last 2 elements are respectively provided by the basic culture medium (e.g., Glascow minimum essential medium, GMEM) and fetal bovine serum (FBS). When provided with all 3 elements, ESCs undergo self-renewing proliferation in a dish and grow into a disorganized mass of SCs, what may resemble an early-stage benign undifferentiated tumor (Fig 3, left). These basic principles apply to most other kinds of SCs that have been successfully cultured, with niche, nutrients, and growth factors requiring optimization for each SC type.

Ever-expanding undifferentiated SC tumors do not normally form in vivo simply because niche signaling is spatially restricted within tissues. Indeed, in a living organism, niche signaling not only ensures SC maintenance, but the size and extent of niche signaling together define a limited space in which SCs can exist [15,16]. When ESCs expanded in vitro are injected back into a blastocyst, even after several passages, control over their proliferation is re-established in vivo, such that ESC tumors do not usually form following their transplantation [9]. In *C*. *elegans*, DTCs have long processes called cytonemes and their extent, along with the level of Notch ligand expression and Notch activation in GSCs, essentially establish the size of the GSC population [54–58]. As proximal SCs are pushed outside of the niche due to the ongoing proliferation within the niche, they progress into their default differentiation path, which lowers their proliferative potential. As such, their ability to initiate an undifferentiated benign tumor becomes highly restricted. Thus, from one standpoint, the niche acts to confine a small undifferentiated SC tumor within a specific region in vivo, essentially preventing it to grow beyond a hard limit.

**Fig 4:**
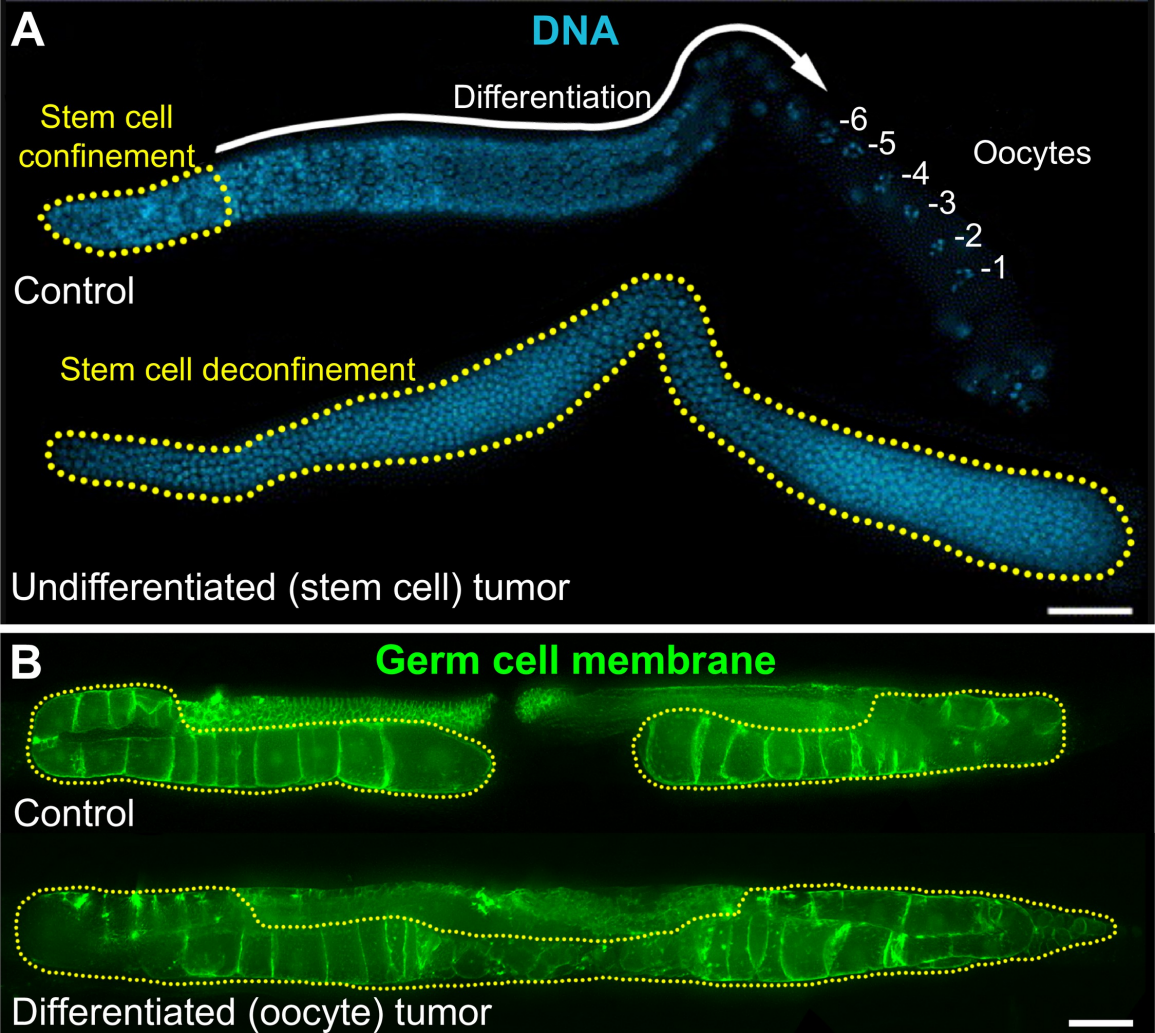
Undifferentiated and differentiated benign germline tumors in *C*. *elegans*. **(A)** Undifferentiated mitotic germ cells (yellow dots) are confined within the niche in wild-type *C*. *elegans* hermaphrodites (top), but not in Notch gain-of-function mutants (bottom). Scale bar, 20 μM. Reproduced with permission [123]. **(B)** When oocytes are not fertilized in *C*. *elegans* hermaphrodites (top), they accumulate up to a certain point (yellow dots), after which homeostatic signaling limits any further SC proliferation and oocyte production. In mutants that disrupt homeostatic signaling however, such as *aak-1*/AMPK null mutants, GSC proliferation and oocyte production do not stop, and if laying is prevented, differentiated benign tumors develop. Scale bar, 50 μM. Reproduced with permission [35]. GSC, germline stem cell; SC, stem cell.

If niche signaling is up-regulated however [59], or if the niche physically expands [60], the region where proliferative SCs are found expands accordingly, and SC numbers increase. If niche signaling becomes fully constitutive, SCs keep proliferating even when they move outside of the niche and form an ever-expanding benign tumor in vivo, very much like they do in a culture dish (Fig 3, right). Spontaneous mutations that activate niche signaling therefore have the potential to induce undifferentiated SC tumors in vivo. In *C*. *elegans*, for example, gain-of-function mutations in the Notch receptor GLP-1 induce the formation of undifferentiated GSC tumors (Fig 4A) [59,61]. Equally, mutations that inactivate genes whose functions are necessary for GSC differentiation can cause the formation of undifferentiated tumors [62–64]. In *Drosophila* male and female germlines, activation of niche signaling, or the inactivation of genes required for GSC differentiation, similarly result in an accumulation of undifferentiated GSCs [65–68]. In the mouse small intestine, SCs are maintained by a Wingless and Int-1 (Wnt) family signal from neighboring Paneth cells, and potentiating that signal either through adenomatous polyposis coli (*Apc*) mutations in intestinal SCs, or by roof plate-specific (R)-spondins, equally promotes SC proliferation, at the expense of differentiation, and result in an expanding intestinal SC compartment [69–74]. Therefore, across most systems, a failure to restrict niche signaling in vivo results in the formation of an undifferentiated SC tumor. In SCs that primarily divide asymmetrically, moreover, loss of polarity regulators can additionally favor symmetrical divisions and promote clonal expansion of the mutated SC [75–77].

Undifferentiated benign tumors are rather seldom found in humans. Although it remains unclear exactly why that is the case, based on how niche signaling works, several reasons may together account for this scarcity. First, germline mutations causing constitutive niche signaling tend to cause dramatic developmental defects and embryonic lethality or sterility. One example is Wnt signaling, a component of hematopoietic and intestinal SC niche signaling, which leads to embryonic lethality in mice when hyperactivated [78,79]. Such mutations must therefore be strongly selected against. Second, single mutations inducing constitutive niche signaling are typically gain-of-function, which are much less frequent than loss-of-function mutations [80]. In addition, mammalian SC niches and niche signals may be more complex than in invertebrates, and may involve multiple cell types and combinatorial niche signals (e.g., hematopoietic SCs [23]), such that single mutations are unlikely to render niche signaling fully constitutive. Third, even though loss-of-function mutations in genes that promote differentiation can also cause undifferentiated SC tumors, these genes typically act redundantly, including in *C*. *elegans* (Fig 1D) [54,64,81], where inactivating mutations in 1 single differentiation gene have little effects on their own. The chances that 2 spontaneous hits occur successively in the 2 (or more) redundant genes in the same SC are much lower.

Another reason for the scarcity of undifferentiated benign tumors in mammals may arise from the fact that many SCs have inherent migratory capacities, like mesenchymal SCs [82,83], such that their expansion may readily represent the development of an invasive lesion.

## Homeostatic stem cell regulation prevents differentiated benign tumors

Mutations in genes encoding several highly conserved proteins prevent homeostatic GSC regulation in *C*. *elegans*. These include loss-of-function in DAF-18/PTEN, PAR-4/LKB1 and AAK-1/AMPK, and a gain-of-function in LET-60/Ras [34,35]. How these genes work together to maintain germline homeostasis is not yet fully understood but homeostatic signal generation is linked to an accumulation of oocytes, the GSC’s terminally differentiated progeny. For this accumulation to happen, oocytes need to arrest in the absence of induction by sperm [84]. This step fails in DAF-18/PTEN, PAR-4/LKB1, and AAK-1/AMPK loss-of-function mutants, as their oocytes spontaneously activate in the absence of sperm signals, are ovulated, and laid as endomitotic oocytes [34,35]. In this case, no terminal cell buildup occurs because unfertilized oocytes are ovulated and laid essentially as quickly as if they were fertilized and laid as eggs, and their turnover time remains normal. However, when the spontaneous activation of oocytes is blocked in *aak-1*/AMPK mutants by the removal of OMA-1 and OMA-2, 2 zinc-finger proteins redundantly required for oocyte activation [85], the deleterious effects of homeostatic signaling failure are far more dramatic. In such animals, GSCs keep proliferating despite the ongoing proximal accumulation of oocytes and a large benign tumor that largely consists of differentiated oocytes, rapidly forms (Fig 4B) [35]. Differentiated benign tumors therefore arise when homeostatic signaling is disrupted. Within tissues in which the terminal cell type is continually expulsed or desquamated at a fast rate, even if SC proliferation were to remain maximal at all times, a benign tumor may not form because terminal cells have a short life expectancy, roughly equal to the inverse of their maximal production rate (e.g., *C*. *elegans* oocytes when sperm is present, or when sperm is absent but DAF-18/PTEN, PAR-4/LKB1, or AAK-1/AMPK are lost). If that life expectancy increases but is not shortly counterbalanced by a decrease in oocyte production (itself enabled by a decrease in GSC proliferation), a benign tumor is expected to grow.

In the *Drosophila* midgut, disruption of homeostatic intestinal SC regulation by ectopic cytokine expression caused an increase in the number of differentiated gut cells, though the tissue remained organized (i.e., hyperplasia) [7,42]. The absence of tumor could however be due to the desquamation of disorganized cells in the gut lumen. In the mammalian intestine, on the other hand, although the disruption of certain tumor suppressor genes, including PTEN, causes the formation of benign differentiated tumors, such as adenomas and hamartomas [74,86–89], whether these gene’s function is to ensure homeostatic regulation of SC proliferation remains to be demonstrated. Nonetheless, assuming this is the case, we conclude that the disruption of homeostatic signaling may result in the formation of differentiated benign tumors across multiple tissues and organisms.

## Basic principles of homeostatic stem cell regulation

**Fig 5:**
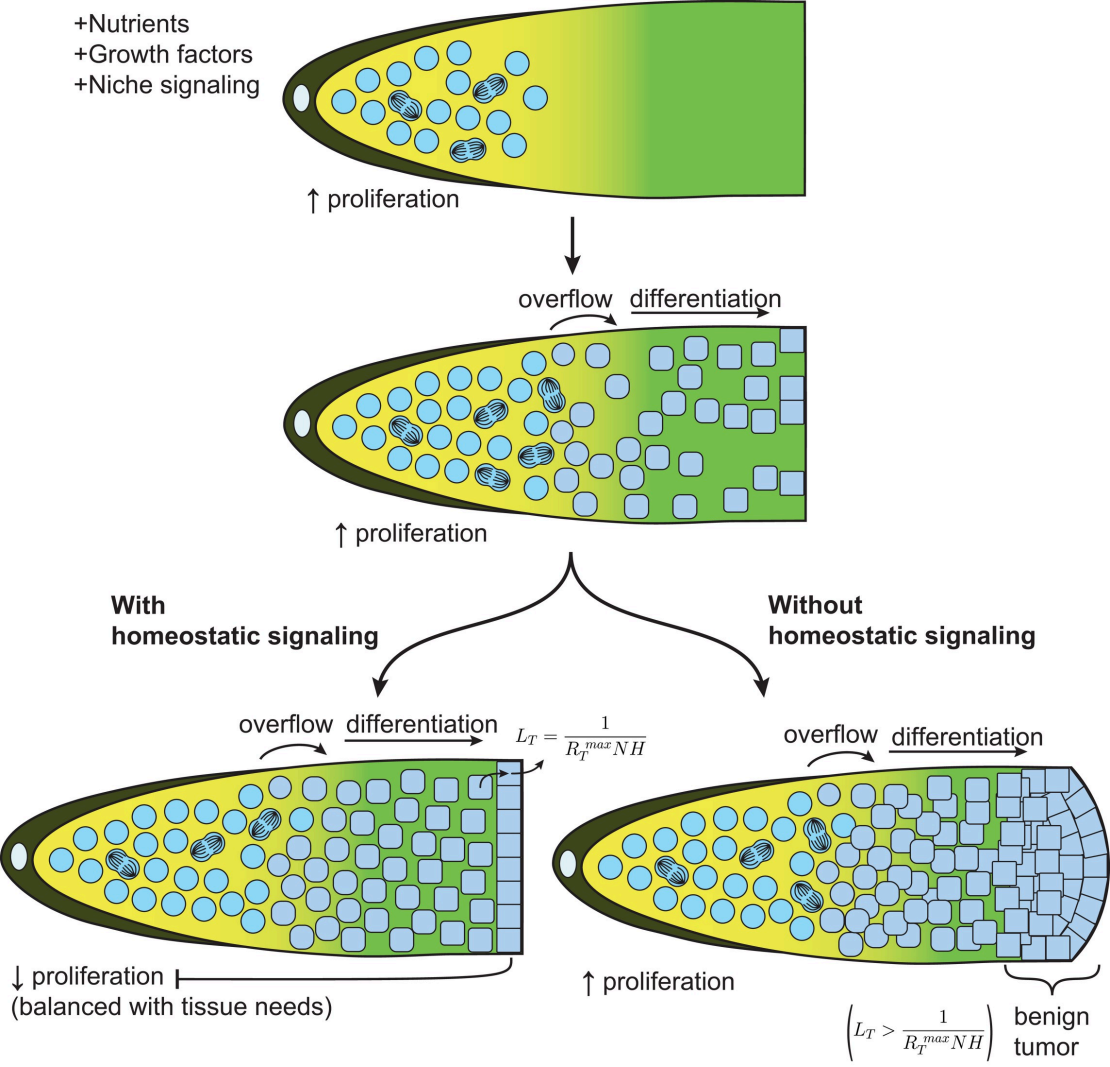
Homeostatic signaling prevents formation of differentiated benign tumors. Top, SCs exposed to niche signaling, growth factors and nutrients, will proliferate to expand in numbers. Middle, Once the niche is filled, SCs begin to overflow outside of the niche and differentiate. Bottom left, Once the tissue has enough terminal cells, homeostatic signaling tunes down SC proliferation. Bottom right, In the absence of homeostatic signaling, SC proliferation always continues at full capacity, causing the formation of a differentiated benign tumor. SC, stem cell.

How does it work? If one considers only niche signaling, growth factors and basic nutrients in an animal that is well nourished, SCs would be expected to proliferate at a high rate until the niche is filled, but to continue to proliferate at the same rate afterwards, generating a continuous overflow of cells spilling outside of the niche (Fig 5, top, middle). Those cells would undergo differentiation and eventually reach a terminal phenotype. For a steady state to exist in the tissue under optimal growth conditions, the terminal cell type life expectancy (L_T_) must be inversely proportionate to the maximal rate (R_T_^max^) at which new terminal cells can be produced (L_T_ = 1/(R_T_^max^N), where N represents a nutritional adjustment factor equal to 1 under optimal growth conditions). As variable environmental conditions can affect the rate of terminal cell production (e.g., starvation) or terminal cell life expectancy (e.g., tissue injuries), to remain balanced, the relationship requires a homeostatic (H) adjustment factor. As such, the role of homeostatic regulation is to prevent the buildup of terminal cells in a tissue, which would inevitably happen under optimal growth conditions in regenerating tissues if terminal cell production was only dependent on nutrition. In other words, if SC proliferation were to depend only on nutrition, all tissues that can regenerate following an injury (which can produce terminal cells faster than they are normally eliminated) would grow differentiated benign tumors in well-fed conditions in the absence of injuries. Accordingly, for all regenerating tissues that do not normally build tumors, L_T_ is expected to be smaller than 1/(R_T_^max^N) and the tissue turnover equation is corrected by an H-factor as follows: L_T_ = 1/(R_T_^max^NH), such that an equilibrium can be maintained (Fig 5, left). On the other hand, in the absence of homeostatic signaling, if terminal cells can be produced faster than they are normally eliminated (i.e., L_T_ > 1/(R_T_^max^N), as in all regenerating tissues), a buildup of terminal cells is expected to occur over time in well-fed organisms (Fig 5, right).

Since the rate of terminal cell production is ultimately dependent on the maximal rate of differentiated progeny production by the underlying stem/progenitor cell population (R_SC_^max^), knowing the relationship between the rate of SC progeny production and terminal cell production allows to predict SC proliferation rates from their terminal cell’s life expectancy at equilibrium, and vice-versa. Namely, R_T_^max^ = XR_SC_^max^, where X is a conversion factor specific for each SC—terminal cell type pair, and L_T_ = 1/(R_T_^max^NH) = 1/(XR_SC_^max^NH). As the relationship between the proliferation of stem cell populations and the life expectancy of their terminal progeny may be buffered by the proliferation of progenitors, and/or pruning, the X factor needs to be adjusted for each SC—terminal cell type pair.

**Fig 6:**
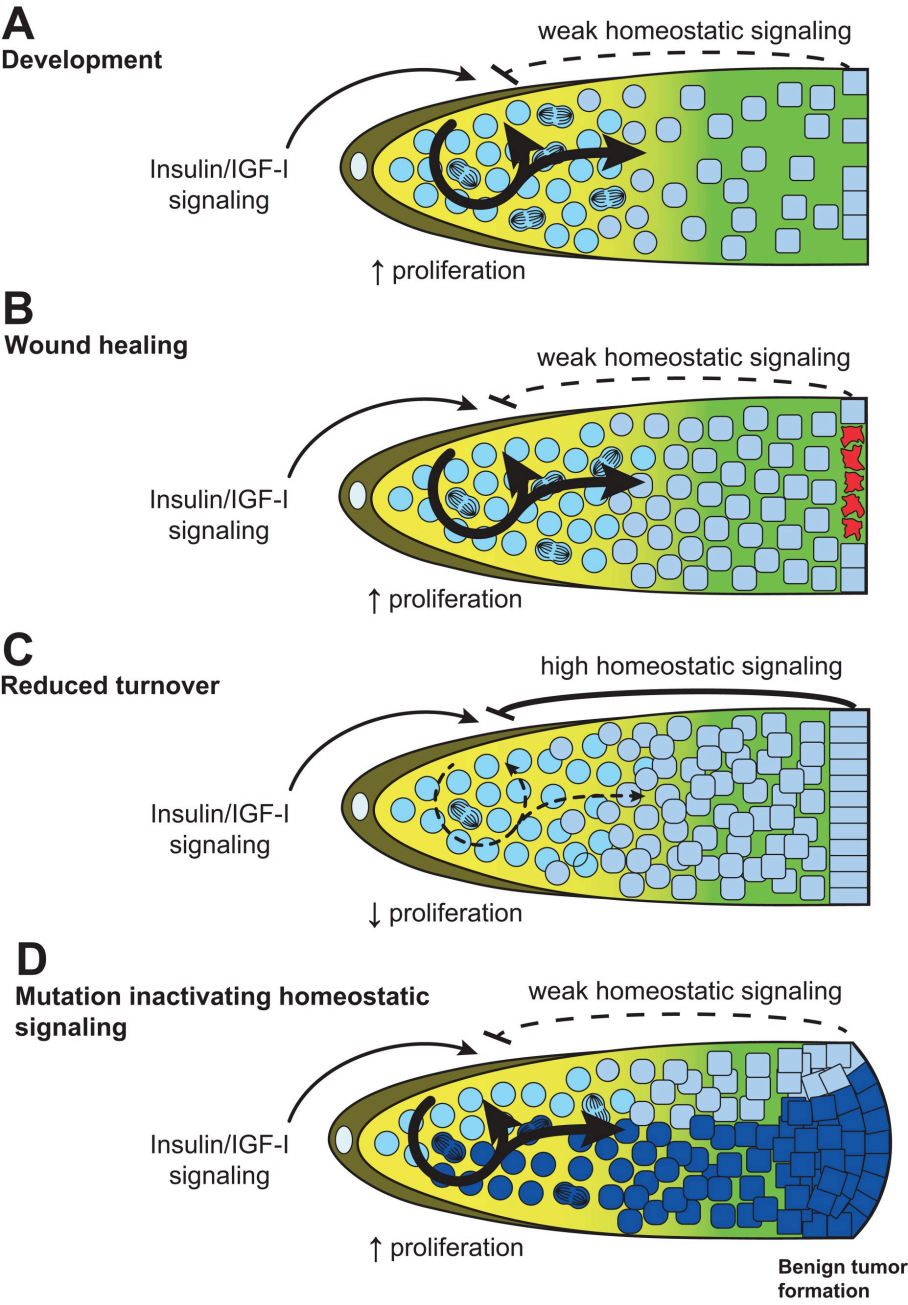
Homeostatic signaling during development and adulthood. **(A)** During development, homeostatic signaling is weak to ensure tissue growth. Stronger homeostatic signaling establishes a steady state during adulthood (see Fig 5, bottom left). **(B)** A wound reduces homeostatic signaling. **(C)** In contrast, if terminal cell turnover is reduced, homeostatic signaling’s strength increases to prevent tumor formation. **(D)** A spontaneous mutation (dark blue shade) disrupting homeostatic signaling undergoes preferential expansion [124] and can lead to the formation of a benign differentiated tumor.

During development or following an injury, the H value may approach 1, allowing SCs to increase proliferation (Fig 6A and 6B). This pushes the actual rate of terminal cell production towards R_T_^max^ and promotes tissue expansion or repair. After completion of the repair, the H-factor must decrease back to its equilibrium value to prevent the overproduction of SC progeny. Conversely, if conditions induce an increase in terminal cell life expectancy, the H-factor must decrease to slow down SC proliferation and prevent terminal cell buildup (Fig 6C). Lastly, a mutation preventing homeostatic regulation would disconnect SC proliferation from tissue needs (H = 1), leaving proliferation rates only governed by nutrition, as during development or a repair situation. However now, in absence of the extra space left either for tissue expansion during development, or to replenish lost cells during tissue repair, the extra terminal cells produced now start to pile up and begin forming an early stage differentiated benign tumor (Fig 6D).

The mammalian intestinal epithelium may be used as an example to extrapolate what such a relationship could mean in more complex tissues and organisms. This epithelium consists of several types of differentiated cells, including enterocytes, goblet and tuft cells, which are generated following the differentiation of transit amplifying cells, themselves generated following intestinal SC proliferation [90]. The differentiated enterocytes that make the inner lining of our intestinal tract have a relatively short life expectancy and must be replaced every 5–7 days, on average, and to replace them, intestinal SCs divide once a day, a relatively high rate, throughout adulthood [91]. The lifespan of an intestinal enterocyte is likely affected by multiple factors, including diet, nutritional status, inflammation, and microbiome constitution. For example, inflammatory bowel conditions are known to accelerate gastrointestinal (GI) epithelial lining desquamation [92], while a range of ingested substances, from irritant laxatives (e.g., bisacodyl) to red meat, may also have similar, perhaps more graded, effects [93,94]. As such, one would expect intestinal SCs to proliferate more in individuals with a higher GI lining turnover. On the other hand, anti-inflammatory treatments may lead to an increase in GI epithelial cell life expectancy in these individuals, while intestinal SC proliferation is expected to decrease accordingly. Assuming such homeostatic regulation takes place in the human GI tract, as in the *Drosophila* intestine [42,95], its disruption is expected to cause the formation of benign intestinal tumors. Such benign tumors would originate from deregulated intestinal SCs, while these deregulated intestinal SCs would show increased proliferation relative to homeostatically-regulated intestinal SCs, and would thus have increased chances of gaining new genetic and epigenetic changes, and of further evolving towards cancer. In support of this view, it was proposed that intestinal cancers originate from intestinal SCs that, through acquiring changes, escaped from normal regulation [96], while patients inflicted with inflammatory bowel diseases have a greater risk of developing polyps [97], and colon cancers [98]. This idea of a progression from a normal intestinal SC to a benign tumor (adenoma) generating intestinal SC finds a strong support in lineage tracing studies performed in the mouse intestine and human colon [2,99]. The idea of a progression from an initially well-differentiated benign tumor, over time, towards a less differentiated and more invasive tumor, is backed by recent genomics analyses [100–102].

## How benign tumors may evolve towards cancer

Once an individual’s reproductive period is over, the effect of natural selection at the organismal level greatly weakens [103] and as a result, the natural selection occurring at the cellular scale gains freedom. At the cellular level, natural selection favors cell proliferation and survival, eventually leading to cancer, which is, unfortunately for the organism, the ultimate fitness status for a cell: immortality and an unlimited proliferation potential. Ironically, many widely used cancer cell lines have won the evolution lottery by getting adopted for laboratory research (e.g., HeLa cells) and are now propagated in multiple laboratories around the globe, having truly achieved immortality. Other more natural examples of highly successful cancer cells include those responsible for the transmissible cancers of dogs, mussels and Tasmanian devils [104,105].

Cancer-initiating mutations are more likely to arise in SCs since they persist and divide throughout our lives [38,106–108]. In contrast, their differentiated progenies all have limited life expectancies and/or proliferation potentials, reducing their evolutionary prospects. As such, SCs uniquely have a higher risk of accumulating genetic and epigenetic changes over the years, and of “evolving” towards cancer. Although this may be less common, early progenitors can undergo transforming mutations conferring them immortality, and can continue to evolve towards cancer thereafter [109,110].

Benign differentiated tumors should therefore typically develop after a SC acquires changes that disrupt homeostatic SC regulation by their differentiated progeny. This could occur, for example, following changes that would prevent proper SC differentiation, such that an accumulation of poorly differentiated SC progeny would no longer promote SC quiescence (Fig 6D). In this case, mutations altering terminal differentiation could promote the formation of benign, partially or aberrantly differentiated tumors. As a result, these mutations increase SC proliferation, and at the same time, they increase SC mutation acquisition rates, and thus accelerate their evolution towards cancer. This phenomenon underlies the exponential clonal expansion of mutant SCs that occurs during aging, something that has been observed in the mammalian hematopoietic system, gut and male germline [111]. In systems in which homeostatic regulation involves multiple tissues, it is possible to imagine situations where deregulation within one tissue would nonautonomously promote the formation of a benign tumor in another tissue.

The transformation of a normal SC into a cancer SC (CSC), that is, one that can propagate a malignant tumor upon transplantation, is not instantaneous and typically occurs over several years in humans. We suppose the multiple intermediate states between the two, showing this progressive deregulation, could be called “pre-cancerous” SCs [112]. The progressive deregulation of a normal SC towards a CSC likely involves several genetic and epigenetic changes leading to the acquisition of the multiple cancer hallmarks [113] within a SC and its clonal progeny. Among these changes, which can occur in any sequence, the escape from niche or homeostatic regulation may constitute major proliferative drivers and be responsible for benign tumor formation.

## Hamartomatous polyposis syndromes could result from homeostatic stem cell deregulation

In humans, hemizygous germline mutations in PTEN or LKB1 lead to Cowden’s and Peutz–Jegher’s syndromes, respectively, which are characterized by the appearance of multiple benign tumors, commonly arising in the GI tract, skin, and mucous membranes, with an age of onset typically between 10 and 30 years old [114,115]. The tumors, called hamartomas, are benign overgrowth largely consisting of differentiated cells [116]. In addition, these patients have a greatly increased lifetime risk for multiple cancers, including GI cancers.

ERK/MAPK is an oncogene that is aberrantly activated in many different types of cancers, including those of the GI tract [117]. Incidentally, work in the *C*. *elegans* germline has shown that PTEN and LKB1 are required for inhibiting ERK/MAPK signaling during homeostatic SC regulation to prevent the formation of differentiated benign tumors [35]. If these principles were conserved in humans, it could explain, at least in part, how Cowden’s and Peutz–Jegher’s patients develop benign tumors, from a problem in homeostatic SC regulation, potentially compromising the down-regulation of ERK/MAPK signaling when there are enough differentiated cells in affected tissues.

In principle, the apparition of hamartomas in Cowden and Peutz–Jegher’s patients could directly result from PTEN and LKB1’s hemizygosity. Tumors typically begin to appear in patients around the onset of adulthood, or after development, when the effect of reduced homeostatic signaling is expected to become more apparent. A subtle reduction in homeostatic signaling, such as one caused by hemizygosity for PTEN or LKB1, would have little consequences during development since tissues are generally expanding, but the effect could become apparent during adulthood, when tissues stop expanding and homeostatic signaling has to be more robust. As such, it may be worth assessing whether a mild inhibition of ERK/MAPK signaling in Cowden’s and Peutz–Jegher’s patients could decrease or delay hamartoma incidence and perhaps even lower cancer risks [118,119].

The increased lifetime cancer risk of these patients can be explained by the increase in proliferation of these patient’s SCs in every tissue where homeostatic regulation relies on PTEN and/or LKB1. Their SCs dividing more frequently than in an average individual, they are indeed more likely to experience a greater number of genetic and epigenetic changes, which, combined with natural selection, are expected to drive overproliferating SCs towards cancer faster and faster, in a vicious circle.

Homozygous PTEN deletions lead to embryonic lethality in mammals [87,120]. Mice were thus created in which PTEN could be specifically deleted in the intestinal epithelium. These mice did not show any obvious perturbation in intestinal SC proliferation or differentiation, but they tended to develop intestinal adenomatous polyps, including some that progressed into cancer [86,88,89]. These results further support the idea that PTEN may act to ensure homeostatic SC regulation in mammals since its loss leads to the formation of benign differentiated tumors.

## Perspectives

Although key tumor suppressors and oncogenes such as PTEN and ERK/MAPK hitherto may play a conserved role in homeostatic signaling across different organisms and stem cell types, some extrapolations presented here remain largely speculative and would require further mechanistic validation in complex models. A range of additional pathways may also be involved in these more complex SC systems. Interestingly, ERK/MAPK promotes SC proliferation cell nonautonomously in *C*. *elegans* from a neighboring tissue [41]. PTEN and LKB1 may therefore also regulate SC proliferation cell nonautonomously through inhibiting ERK/MAPK in this tissue. Homeostatic regulation may similarly involve several cell types in humans, and it will certainly be a challenge to solve it down to the cell and molecular levels for every SC systems. However, the resulting knowledge should greatly facilitate cancer prevention and treatment, as well as all SC-based therapies.
